# Impact of Anatomical Research Projects for Medical Students: A Cross‐Sectional Survey of Academic and Professional Skills, Clinical Aspirations and Appreciation of Anatomy

**DOI:** 10.1002/ca.24259

**Published:** 2025-01-19

**Authors:** Amil Sinha, Arun James Thirunavukarasu, Anosh Bonshahi, Cecilia Brassett

**Affiliations:** ^1^ Human Anatomy Centre, Department of Physiology, Development and Neuroscience University of Cambridge Cambridge UK; ^2^ New Cross Hospital Royal Wolverhampton NHS Trust Wolverhampton UK; ^3^ Oxford University Clinical Academic Graduate School University of Oxford Oxford UK; ^4^ Stepping Hill Hospital Stockport NHS Foundation Trust Stockport UK

**Keywords:** anatomical research, cadaveric, dissection, education, medical school, medical student, training

## Abstract

In the third year of pre‐clinical medicine (known as Part II of the Natural Sciences Tripos at the University of Cambridge), students have the opportunity to lead a primary research project on clinically relevant anatomy, often involving donor dissection. This descriptive study used a cross‐sectional survey to explore the effects of undertaking an anatomical research project on students' attitudes, interests, and a variety of academic and professional skills. Of 45 students who were invited to participate in this study, 40 responded. Of these, 30 students (75%) had performed cadaveric dissection. Projects increased students' interests in academic careers (36% or 90% agreed/strongly agreed) and scientific inquiry, with 30 students (75%) undertaking subsequent research. Many students (30/40; 75%) strongly agreed their projects highlighted the importance of considering the scientific literature when providing patient care. Most (39/40; 97.5%) felt that there was scope for further anatomical research to appreciate and explore anatomical variation. Many students (32/40; 80%) strongly agreed that projects improved their self‐directed learning skills. Inductive thematic analysis of free‐text answers identified themes of improved academic, practical, and professional skills such as negotiation, responding to questions, presenting at conferences, and liaising with experts and non‐experts. These results suggest that anatomical primary research through this program effectively fosters academic aptitude and interest, as well as the practical and professional skills necessary to thrive in academia and clinical medicine. Aspirations for a surgical career were strengthened and valuable anatomical context was provided.

## 1 Introduction

Students are taught anatomy in a variety of formats at medical schools around the world. Teaching formats range from didactic lectures to problem‐based learning, self‐study of theory to hands‐on experience, and examination‐focused curricula with summative assessment to elective periods with student‐directed practical sessions. Traditionally, anatomy has been taught through donor‐based dissection and lectures, although these patterns have begun to change in recent years (Estai and Bunt 2016; Iwanaga et al. 2021). Specifically, interaction with prosections has become a popular alternative to cadaveric dissection, with plastinated specimens being often used (Iwanaga et al. 2021). Further innovative pedagogical techniques have benefited from technological developments, including videos, 3D printing, web‐based resources, and virtual reality (Iwanaga et al. 2021). Although some exposure to primary research has been shown to improve student's confidence in their academic ability (Knight and Ma 2018), medical students rarely have the opportunity to participate in anatomical research and the accompanying exposure to controversies, latest findings, and skills acquisition. Despite these problems, there is a paucity of literature relating to anatomical research led by medical students and their perspectives.

In addition to teaching students the “basis of the medical sciences,” anatomy education can also have a significant impact on students' future career choices and success (Smith and Mathias 2011). Most doctors consider their anatomical education to be invaluable in clinical practice, particularly in radiology and surgery, and anatomy teaching (especially courses that incorporate cadaveric dissection) increases the likelihood of applying for and successfully securing surgical jobs in the future (Smith and Mathias 2011; Pulcrano, Malekzadeh, and Kumar 2016; Santini et al. 2019; Schroeder et al. 2020; Zhang et al. 2020). As students lost access to much in‐person anatomy teaching during the COVID‐19 pandemic, interest and aptitude for surgical careers may have been significantly impacted (Iwanaga et al. 2021; Franchi 2020). It is therefore important to provide effective anatomy teaching with opportunities for supplemental anatomy projects, to nurture competent future surgeons.

At the University of Cambridge, third‐year medical students undertake a compulsory “intercalation” year in a Tripos (BA degree course) of their choosing, known as the Part II year of their degree. The term Part II, as opposed to the previous 2 years (Part IA and Part IB) which mainly comprise taught courses is used to indicate a research‐oriented year of in‐depth study into a specialist subject. For those offered a place in the “Department of Physiology, Development, and Neuroscience,” a number of anatomical research projects are available, which contribute to their final degree class. Anatomy‐based research projects have been made available since academic year 2014–15 and have been offered annually since then to an average of six students each year. Students undertake an independent research project supervised by senior anatomists and clinicians. Supervisors hold preliminary discussions with students to outline an overall direction for the project. Students are then encouraged to take ownership of their work and proactively shape the project as they learn more through extensive literature searches, discussions with specialists in the field, and practical findings from work with volunteers or cadaveric dissection. Through this work, students develop the required skills to develop and test hypotheses, conduct and report data analysis, and engage with various data collection tools ranging from imaging and 3D reconstructions to histopathology. The project spans the entire academic year, providing time for students to trial ideas and build on experiences.

The processes of self‐reflection, mentor input, and conducting literature reviews facilitate the cognitive constructivist model of educational pedagogy to promote personal and academic growth (Powell and Kalina 2009). Furthermore, exposure to academically social environments in the dissection room, hospital, and laboratories promotes social and cultural interactions with fellow students, technicians, and senior mentors, which can act as triggering mechanisms for learning; a foundational principle of social constructivist theory (Powell and Kalina 2009; Driscoll 2005). In addition to nurturing the growth of academic minds, multiple anatomy‐based research projects have applications to clinical practice, such as developing an ultrasound protocol for imaging the temporomandibular joint, delineating anatomical variation in nerves, which are vulnerable in certain surgical procedures, and defining cephalometric parameters to aid planning of orthognathic surgery (Chu et al. 2023; Thirunavukarasu et al. 2021; Bonshahi et al. 2021).

These projects encompass a diverse range of surgical and interventional specialties intending to address clinical challenges or areas of interest in each. The majority of these research projects involve cadaveric dissection to facilitate primary anatomical research through active dissection, experimentation, and direct comparison between specimens. Thus, the projects aim to contribute to current clinical understanding and practice while nurturing the academic and practical skills doctors require. A small number of projects have been focused on the theoretical and historical aspects of dissection itself, examining how anatomical study has evolved through time since the illustrations of Leonardo Da Vinci and Andreas Vesalius.

Anatomical research projects thereby aim to foster interest and aptitude in anatomy, while creating strong foundational skills to support students in their future academic and clinical endeavors, particularly relating to the surgical specialties. However, the degree to which these projects are effective in achieving these aims is not well‐understood. This descriptive study explored whether these aims are fulfilled by anatomy research projects. Specifically, we invited previous anatomy students to answer questions to investigate the perceived effects of completing research projects on their career aspirations, academic and professional skills, and appreciation of anatomy.

## 2 Methods

### 2.1 Ethical Approval

The protocol for this study was approved by the Cambridge Higher Education Studies Research Ethics Committee (CHESREC) with the code: CHESREC.2023.ET.55.Sinha.

### 2.2 Survey

Emails were distributed to students who had completed Part II anatomical projects at the University of Cambridge between the academic years 2014–2015 and 2022–2023 to invite them to participate in this study. Averages of six students each year were accepted onto the program. Students who completed projects focused on art, history, or theoretical aspects of dissection were excluded. Included within this email (Supporting Information 1) was a link to the survey (Supporting Information 2), a participant information sheet (PIS, Supporting Information 3), and a consent form (Supporting Information 4). Participants could only submit responses after they had read the PIS and provided consent. They completed the survey online in their own time and were prompted to respond three times over 2 months.

The survey was structured to enquire about the impact on four domains: academia, clinical aspirations, appreciation of anatomy, and professional skills. Each of these four domains comprised a combination of closed questions using Likert scales and open questions for free‐text answers, which enabled both quantitative and qualitative analysis.

### 2.3 Analysis

Data was collected using a 5‐point Likert scale with the following anchor statements: strongly disagree, disagree, neutral, agree, strongly agree. These were presented graphically and interpreted quantitatively using relative proportions. Appraisal of questions with yes/no/maybe options was based on relative proportions—imbalances of positive and negative, and indeterminate answers. To establish whether there were significant differences in the participants' clinical aspirations before and after undertaking an anatomy project, the Chi‐squared test was used. Free‐text answers were analyzed through a process of inductive thematic analysis undertaken by a single researcher (AS), with their output appraised by two other researchers (AJT and AB) to gauge accuracy. Through this process, key themes and sub‐themes were described by identifying commonly used or pertinent terms and phrases. Analysis and data visualization were conducted with Microsoft Excel for Mac (version 16.57; Microsoft Corporation, Redmond, Washington, United States) and R (version 4.1.2; R Foundation for Statistical Computing, Vienna, Austria).

## 3 Results

**FIGURE 1:**
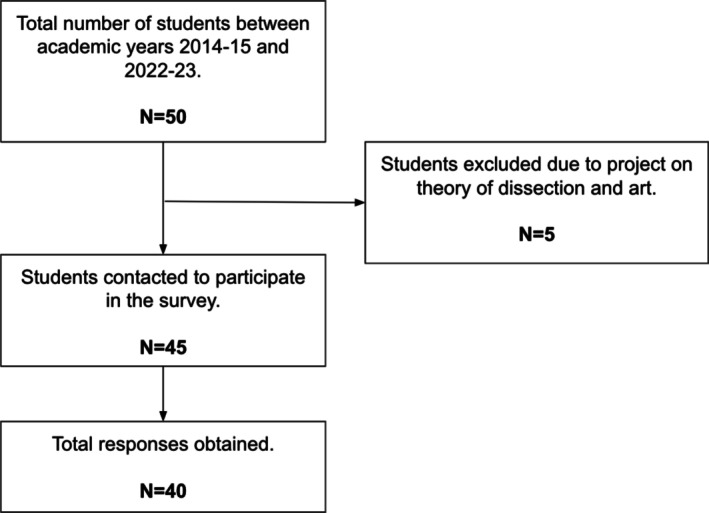
Flow chart depicting how participants were selected. Of 50 individuals who had undertaken Part II anatomical research projects, 5 were excluded for work related to art, history, or theoretical aspects of dissection. Of the 45 participants invited to participate in the study, 40 responded and were included in subsequent analysis.

A total of 50 students participated in Part II anatomy research projects between the academic years 2014–2015 and 2022–2023; 5 were excluded as their projects were primarily based on art, history, or theoretical aspects of dissection. Therefore, 45 students were invited to participate in the study and 40 responses were received: a participation rate of 88.9% (Figure 1). Of these 40 projects, 32 (80.0%) related to a specific clinical specialty. These specialties were clinical radiology (*n* = 1); otorhinolaryngology (*n* = 2); gastroenterology (*n* = 6); oral and maxillofacial surgery (*n* = 5); plastic surgery (*n* = 2); regional anesthesia (*n* = 1); and trauma and orthopedics (*n* = 15). Thirty projects (75.0%) involved cadaveric dissection. The 10 projects (25%) that did not involve cadaveric dissection were related to radiology within otorhinolaryngology, trauma, and orthopedics as well as oral and maxillofacial surgery.

### 3.1 Influence on Attitudes to Academia

**FIGURE 2:**
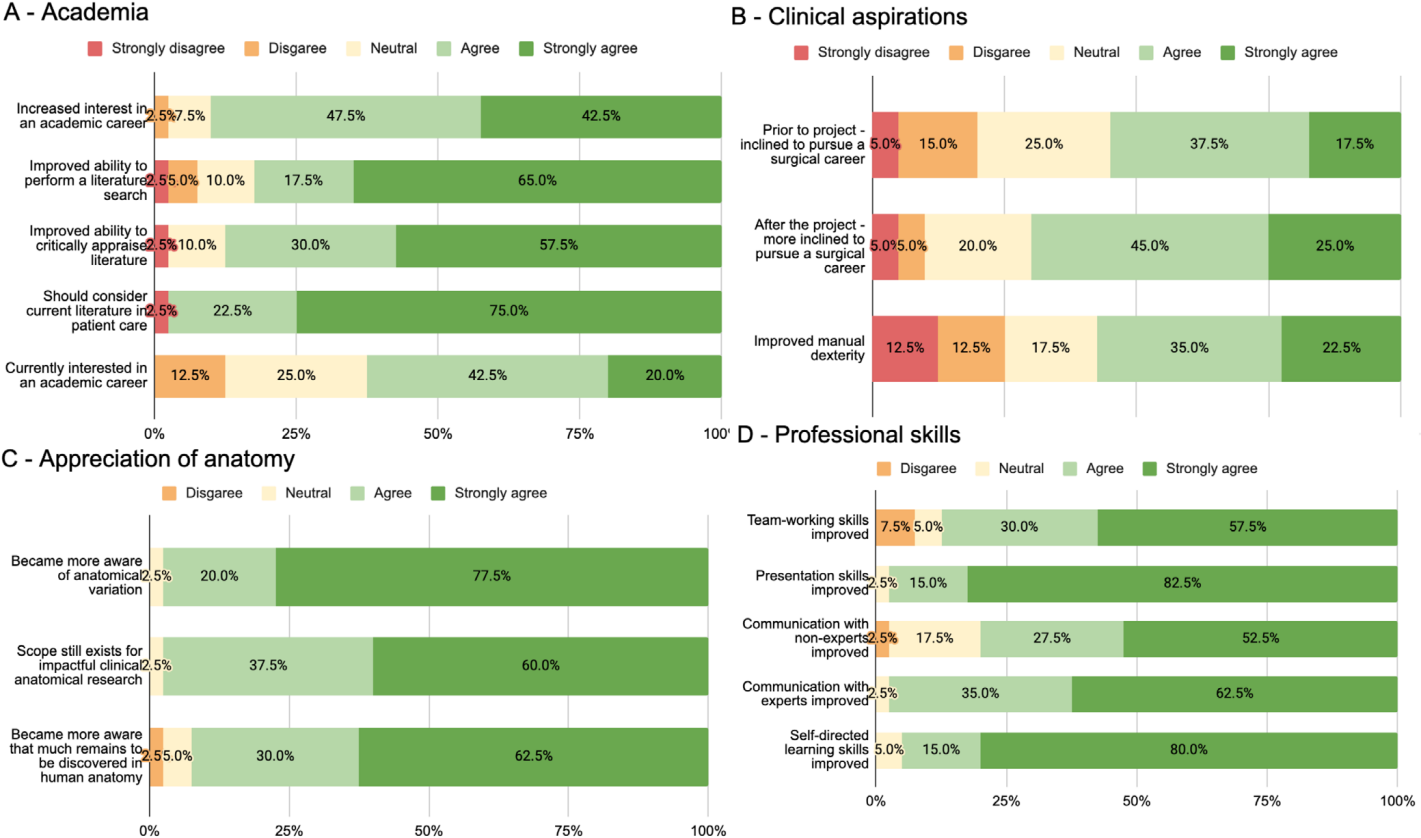
Responses to Likert scales. Total responses *N* = 40. These results indicate that the majority of students agree or strongly agree that completing this project had a positive impact on their academic ability (A), appreciation of anatomy (C) and professional skills (D). The proportion of students who were more inclined to pursue a surgical career increased after completing the project (B).

**FIGURE 3:**
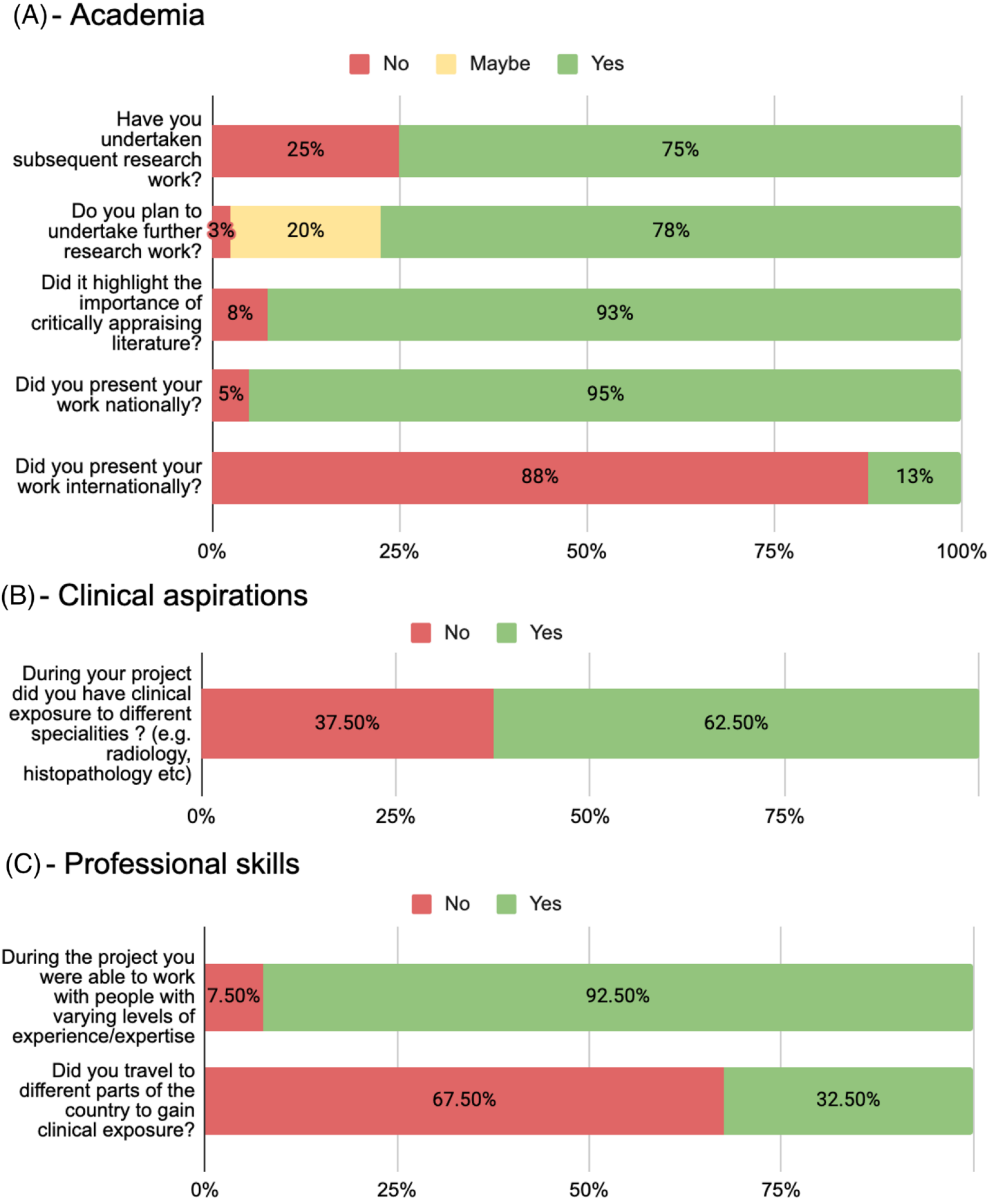
Responses to questions using yes‐no‐maybe answers. (A) = Academia; (B) = Clinical aspirations; (C) = Professional skills. Total responses *N* = 40. These results highlight that the majority of students have presented their work at conferences and have done further research work. Projects also provided opportunities to work with people of varying experience/expertise.

Responses to questions relating to academia are summarized in Figures 2A and 3A. 90% (36/40) of respondents agreed (47.5%) or strongly agreed (42.5%) that completing the project increased their interest in an academic career, with the majority (75% or 30/40 respondents) having undertaken subsequent research work. 77.5% (31/40) plan to undertake further research work. Respondents generally felt that their projects had improved their ability to perform literature searches (82.5% or 33/40 agreed or strongly agreed) and critically appraise literature (87.5% or 35/40 agreed or strongly agreed). Almost all respondents (92.5% or 37/40) thought their projects had highlighted the importance of critical evaluation of previous research, and almost all felt it was important to consider current literature in patient management (97.5% or 39/40 agreed or strongly agreed). In terms of achievement, most students had presented work from their projects nationally (95% or 38/40), and 12.5% (5/40) had given international presentations.

### 3.2 Influence on Clinical Aspirations

Responses relating to the clinical aspirations of students are summarized in Figures 2B and 3B. The overall rate of surgical aspiration increased after students had undertaken anatomy projects: While, the number of participants who aimed to pursue a surgical career rose from 22 (55%) before to 28 (70%) after their project, this modest increase did not reach statistical significance (Chi‐squared test = 1.92, *p* = 0.16). Most students (62.5% or 25) were exposed to different specialties during their project (Figure 3B).

### 3.3 Influence on the Appreciation of Anatomy

Responses to questions relating to students' appreciation of anatomy are summarized in Figure 2C. Almost all participants felt that their projects had made them more aware of anatomical variation (97.5% or 39/40) and of knowledge gaps requiring further anatomical research (92.5% or 37/40). Almost all students (97.5% or 39/40) agreed or strongly agreed that there was still scope for impactful research in clinical anatomy, with the single remaining response being neutral.

### 3.4 Influence on Professional Skills

Responses relating to non‐technical, professional skills are summarized in Figures 2D and 3C. Of note, 97.5% (39/40) of respondents agreed or strongly agreed that completing the project had improved their presentation skills as well as communication with experts. Furthermore, 95% (38/40) of respondents agreed (15%) or strongly agreed (80%) that their self‐directed learning skills had improved. Similarly, completing the project had led to an improvement in teamwork skills (87.5% agree/strongly agree) and communication with non‐experts (80% agree/strongly agree). Non‐experts referred to people who were not specifically working in the field of the project, and included near‐peers, junior medical students, laboratory technicians and doctors from other specialties. Approximately one‐third (32.5%) of respondents had gained further clinical exposure by attending theaters, clinics, and wards in hospitals in different parts of the country (Figure 3C).

### 3.5 Inductive Thematic Analysis

Qualitative responses to the open questions within each outcome category were analyzed using reflexive thematic analysis with an inductive approach. The main themes identified and defined were:

1. Academic skills—Gaining experience in experimental and clinical research in addition to the practice of key skills including planning, data collection, analysis, discussion, and academic writing.
2. Practical skills—Learning both physical skills, such as fine dissection and using software tools for imaging, cadaveric study, and data analysis
3. Professional skills—Communication in different settings including presenting, negotiating, responding to comments, and liaising with people of different expertise and backgrounds.

Words and phrases repeated multiple times or representing unique responses were collated under a set of sub‐themes within the above themes. Almost all of these free‐text responses had a positive attitude, with only one respondent stating, “I cannot say that I improved in my practical skills”. This is summarized in Table 1.

**TABLE 1:** Thematic analysis stating the number of responses that included the terms and phrases related to the sub‐themes and themes. Three main themes were identified: Academic skills, practical skills, and professional skills. No negative phrases were identified.

Theme	Academic		
Sub‐themes	Subsequent research work	Gain from presenting work	Traveling to different parts of the country
Terms (frequency)	– Continuing the same project/another anatomy project (2) – Surgical‐based project (8) – Clinical project (did not specify whether surgical) (15) – Publications: Randomized controlled trials, Systematic reviews, Literature reviews, Audits, Quality improvement projects, Write‐ups, Case reports (7)	– Confidence (12) – CV boost (2) – Experience making presentations and abstracts (16)	– Experience of a different clinical environment (9) – Greater appreciation of anatomy and its relevance (1)

Theme	Practical skills	
Sub‐themes	Types of practical skills improved	Traveling to different parts of the country
Terms (frequency)	– Dissection—fine, blunt, microsurgery (27) – Software skills—coding, digitizing, stats (11) – Imaging and image analysis (CT, MRI, US)—(11) – Histopathology (1)	– Further practical skills—assisting in theater, dissection, ultrasound (3)

Theme	Professional skills	
Sub‐themes	Gain from presenting work	Non‐technical skills
Terms (frequency)	– Communications skills (presenting, fielding questions, rationalizing) (30) – Networking with like‐minded individuals (2)	– Liaising and collaborating with other professionals (peers, seniors, dissection room staff) (39) – Communicating/recruiting volunteers/patients (4) – Personal skills developed—negotiation, decision‐making, leadership, managing uncertainty, communication, problem solving and proactively using feedback, online meetings (12) – Prepared me very well for healthcare work in a multidisciplinary team (1)

## 4 Discussion

The Medical School Council (MSC) outlines a range of attributes derived from National Health Service (NHS) values that medical students are expected to demonstrate (Search|Medical Schools Council 2024). These include academic and problem‐solving ability, effective communication and teamwork, personal organization, and the ability to reflect on one's strengths and weaknesses. These are also reflected in the “The Duties of a Doctor:” a definitive exposition concerning how good doctors should behave, published by the General Medical Council (The duties of a doctor registered with the General Medical Council 2024). *The Duties of a Doctor* states that doctors should:

1. Keep their professional knowledge and skills up to date and work within the limits of their competence.
2. Work in partnership with patients and colleagues, respecting their dignity and interests.
3. Always be open and act with integrity, never discriminating between patients or colleagues.
4. Take prompt action if you think that patient safety, dignity, or comfort is being compromised.

The effects of leading an anatomical research project on the four domains assessed by this study overlap with the expectations set by the MSC and GMC. Thus, assessing these outcomes is valuable in investigating how conducting primary research fosters the skills necessary in clinical as well as academic medicine.

Our survey results using Likert scales highlight that most students felt their key academic skills had improved, including performing literature searches, critically appraising existing literature, and increasing awareness of the importance of considering current literature when delivering patient care. Multiple free‐text responses explained that as this project was their first experience of leading and presenting academic work, it contributed towards forming a foundation of skills and knowledge on which they could build. Three‐quarters of students have subsequently undertaken further academic research, with more planning on doing so. Thematic analysis of free‐text responses showed that this subsequent research had been undertaken in various formats, ranging from audits and quality improvement projects (QIPs), through to systematic reviews (SRs) and randomized control trials (RCTs).

Projects not only provided an introduction to the practicalities of primary research, but also nurtured professional skills required for success in both the academic and clinical environments of medicine. Presenting their work increased confidence in presenting their data and conclusions, responding under pressure to questioning, and explaining their study rationale to experts and non‐experts. Furthermore, almost all respondents stated that their presentation skills had improved, and that liaising and collaborating with other professionals had significantly enhanced their communication skills, preparing them well for working in multi‐disciplinary teams in clinical practice. Of particular interest was the fact that 95% of respondents (80% strongly agree, 15% agree) felt that completing this project had significantly improved their self‐directed learning (SDL) skills. These skills are widely considered to be especially important, as lifelong learning is integral to keeping skills and knowledge up to date and thus optimizing patient care using an evidence‐based approach (Murad et al. 2010). Knowles describes the essential components of SDL including: an educator facilitating learning rather than merely acting as a source of content; learners working with educators to identify their learning needs, objectives and resources; learners actively implementing the learning process; learners committed to a learning contract with regular evaluation (Knowles 1975). The structure of Part II anatomical research projects involves an expert within the field, typically a consultant physician or surgeon, and students work with them to adapt and develop their project, working towards a mandated summative dissertation but also with a view to presenting and publishing their work. All these elements fulfill the components described by Knowles, and may explain why almost all students felt the project had improved their SDL skills. SDL as adult learners becomes more integral to career progression and self‐development as one becomes more senior within the medical field and is clearly stipulated in the GMC document “The Duties of a Doctor” (The duties of a doctor registered with the General Medical Council 2024). This draws on Kolb's reflective cycle of experiential learning, which consists of a 4‐stage cycle of concrete experience, reflective observation, abstract conceptualization, and active experimentation (Kolb 1984). These projects require students to engage in this process and thus allow these skills to be nurtured from an early stage, facilitating their progression into post‐graduate medicine and education where this process is necessary to maintain and deliver a high standard of patient care.

Although the proportion of students who were inclined to pursue a career in surgery had increased after completing the project, this was not statistically significant. This is likely to be a consequence of the high pre‐project interest in surgery, as anatomical projects were typically related to surgical specialties. Nonetheless, our results indicate that projects had reinforced students' interest in surgery. Students also developed a greater appreciation of anatomy, becoming more aware of anatomical variation and realizing the scope for impactful clinical anatomical research. No respondents disagreed with those statements. Additionally, while 75% of responses stated that their project involved cadaveric dissection, 57.5% agreed or strongly agreed that their manual dexterity had improved because of their work. These findings show that completing these projects supports surgically‐minded medical students in gaining valuable exposure to and understanding of anatomical variation, with a greater appreciation of its clinical importance. This was facilitated by access to multiple cadaveric specimens, prosections, and clinical imaging. The process of cadaveric dissection supports the transference of the concept of anatomical variation from a theoretical concept into concrete reality for students (Ghosh 2017). Furthermore, in addition to developing the practical skill of dissection, results from the thematic analysis identified the development of other skills, such as proficiency in using new digital software, data visualization, and statistical analysis: invaluable skills for academic and clinical doctors. This was especially evident in the responses from students who did not perform cadaveric dissection (25%). These projects focused on clinically relevant radiology within surgical specialties and although this did not facilitate the acquisition of practical dissection skills, it supported the capabilities of image interpretation and analysis, vital skills surgeons use daily. The likert scale responses from these students did not show a significant deviation from the rest of the cohort apart from in response to the question “You are interested in an academic career in the future”, where 3 out of these 10 students (30%) disagreed. Out of the remaining cohort whose projects did involve cadaveric dissection, a total of 2 out of these 30 students (6.67%) disagreed with this statement. This could suggest that students enjoyed the practical aspect of academia more, however, the limited sample size and data collected to this effect makes it difficult to draw conclusions.

Overall, the results suggest that students perceive primary anatomical research to foster the knowledge, interest, and skills necessary to undertake academic clinical work in the future, particularly in surgical specialties. Moreover, students who undertook anatomy projects developed a greater appreciation for clinical anatomy as an academic discipline with relevance to medical and surgical practice. Students exhibited significant output in terms of national and international presentations (Figure 3A), as well as peer‐reviewed publications (Table 1). Participants generally agreed that their professional skills had improved because of their projects. This process enabled students to gain valuable exposure to the academic world of medicine, with 90% (47.5% agree, 42.5% strongly agree) of respondents stating that the project had increased their interest in an academic career.

### 4.1 Limitations

This study was limited by three main factors. First, while the response rate was high, it is possible that students who were enthusiastic about the program were more likely to respond. Second, as students apply for a limited number of anatomy projects each year, and as participants were already inclined to pursue surgery before undertaking their anatomical projects, the surveyed cohort likely had more interest in academic anatomy and surgery than medical students as a broader group. Future comparative studies aiming to establish the effect of a program like this may control for this confounder through comparisons with other types of projects and subjects; and by adjusting for previous and subsequent attainment. Third, this retrospective study prohibits definitive conclusions about causality, and reduces confidence in results derived from questions relating to opinions before participants had undertaken an anatomical project. Subsequent survey studies could employ a before‐after design to further explore the effects of anatomical research projects on students' aspirations, knowledge, and skills.

### 4.2 Directions for Further Work

To definitively establish the effect size of anatomical research projects, prospective survey studies could be undertaken with before‐after or comparative designs using attainment‐matched students in other courses. Specific work is indicated to establish the types of students in which anatomical research projects will produce the positive effects detailed above. Ideally, access to such projects should be broadened to all those who would benefit, although this would require considerable investment of resources in terms of time, materials, and relevant experts who would be able to act as supervisors.

## 5 Conclusions

Completion of Part II Anatomical Research Projects in the third‐year of preclinical studies at the University of Cambridge results in perceived significant positive impact on building foundational academic and professional skills for medical students who pursue a clinical or academic career path. Specifically, it reinforces an appreciation for anatomy and aspirations for a surgical career, which are both inextricably linked. Anatomy projects therefore seem to promote the qualities of a good doctor as defined by the MSC and GMC in “The Duties of a Doctor.” Other medical schools may wish to consider the establishment of similar projects to support the development of these key skills.

## Ethics Statement

Granted by the Cambridge Higher Education Studies Research Ethics Committee (CHESREC), Ethics Code: CHESREC.2023.ET.55.Sinha.

## Consent

No patients were involved in this survey study following a protocol with ethical approval.

## Supporting information


Data S1.

Data S2.

Data S3.

Data S4.

## Data Availability

The raw data reported in this paper can be provided upon request.
