# Supplementary material for: Impact of Anatomical Research Projects for Medical Students: A Cross‐Sectional Survey of Academic and Professional Skills, Clinical Aspirations and Appreciation of Anatomy
Source: Clin Anat. 2025 Jan 19;38(3):347–54. doi: 10.1002/ca.24259 (PMC11925134; doi:10.1002/ca.24259)
Supplement: Supplementary file 1 — Data S1. [file CA-38-347-s003.docx]

Recruitment Document

- The following email shall be sent to students who meet the inclusion criteria for the study:
  - Having completed a clinical anatomy-based Part 2 project at the University of Cambridge during the period 2014-15 and 2022-23.
- All email addresses shall be ‘BCC’d’ to ensure no breach of confidentiality.
- Attached to this email shall be three documents:
  - Participant information sheet
  - Consent form
  - Survey

—-----------------------------------------------------------------------

*Dear all,*

*Hope you are keeping well!*

*I am emailing on behalf of the Part 2 Anatomy Team as we understand that you completed a Part 2 Anatomy Project during your intercalation year at the University of Cambridge.*

*The team were keen to gather some information about your experience of doing a Part 2 Anatomy Project as part of a study. This can help us understand the impact this had on your inter-personal skills, career goals as well as academic and clinical acumen. Furthermore, your responses shall be used to inform future developments in the programme. Please read the attached ‘Participation information sheet’ and ‘consent form’ for further information.*

*After reading this, if you are happy to participate, we would be very grateful for your contribution and request that you complete the following short survey (5-10 minutes) as accurately as you can:*

*https://forms.gle/T8Sa4ovJcW1Apxqz6*

*Thank you for taking the time to complete this! :)*

*Kind regards,*

*Dr Amil Sinha*

*MB BChir, MA (Cantab)*

—-----------------------------------------------------------------------
