# Supplementary material for: Impact of Anatomical Research Projects for Medical Students: A Cross‐Sectional Survey of Academic and Professional Skills, Clinical Aspirations and Appreciation of Anatomy
Source: Clin Anat. 2025 Jan 19;38(3):347–54. doi: 10.1002/ca.24259 (PMC11925134; doi:10.1002/ca.24259)
Supplement: Supplementary file 2 — Data S2. [file CA-38-347-s001.docx]

Part 2 Anatomy Project Feedback Survey

Dear all,

I hope that you all are keeping safe and are well!

Disclaimer:

All of your responses will be anonymised and stored in a secure password-protected file. Your anonymised responses shall be used to inform future developments in the programme as well as in potential presentations or publications about Part 2 Anatomy. Please read through the Participant Information Sheet (PIS) and consent form attached to the recruitment email.

Thank you for taking the time to complete this survey.

There are a total of 5 sections and the whole survey should take approximately 5-10 minutes to complete.

We greatly appreciate your honest feedback about your experience of doing a Part 2 Anatomy Project!

We wish you the best for the future!

* = required answer.

I have read the participant information sheet and consent document provided and provide my consent to participate.*

- I consent

Section 1 Demographics:

Select the stage of your medical career*

1. 3rd Year Medical Student
2. 4th Year Medical Student
3. 5th Year Medical Student
4. 6th Year Medical Student
5. Foundation Year 1
6. Foundation Year 2
7. Foundation year 3
8. Internal Medicine Training
9. Core Surgical Training
10. Specialist Medical Training
11. Speciality Surgical Training
12. GP training

Was your project related to a clinical specialty?*

- Yes, No

If you answered 'Yes' above, please state it here.

- *Free Text Answer*

Did your project involve cadaveric dissection?*

- Yes, No

Section 2 Academia:

You are interested in an academic career in the future*

- Strongly Disagree, Disagree, Neutral, Agree, Strongly Agree

The project experience increased/positively impacted your interest in an academic career*

- Strongly Disagree, Disagree, Neutral, Agree, Strongly Agree

Have you undertaken subsequent research work?*

- Yes, No

If you answered 'Yes' above, please provide brief details.

- *Free Text Answer*

Do you plan to undertake further research work?*

- Yes, No, Maybe

It improved your ability to perform a literature search*

- Strongly Disagree, Disagree, Neutral, Agree, Strongly Agree

It improved your ability to critically appraise literature*

- Strongly Disagree, Disagree, Neutral, Agree, Strongly Agree

Did it highlight the importance of critically appraising literature?*

- Yes, No

It is important to consider current literature in patient management*

- Strongly Disagree, Disagree, Neutral, Agree, Strongly Agree

Did you present your work nationally?*

- Yes, No

If you answered 'Yes' above, please provide brief details.

- *Free Text Answer*

Did you present your work internationally?*

- Yes, No

If you answered 'Yes' above, please provide brief details.

- *Free Text Answer*

How long after your project did you present your work?

- *Free Text Answer*

What did you gain from the experience of presenting your work?

- *Free Text Answer*

Section 3 Clinical Aspirations:

Prior to starting the project were you more inclined to pursue a surgical rather than medical career*

- Strongly Disagree, Disagree, Neutral, Agree, Strongly Agree

After completing your project were you more inclined to pursue a surgical rather than medical career*

- Strongly Disagree, Disagree, Neutral, Agree, Strongly Agree

During your project did you have clinical exposure to different specialities ? (e.g. radiology, histopathology etc)*

- Yes, No

If you answered 'Yes' above, please provide brief details.

- *Free Text Answer*

The project improved your manual dexterity*

- Strongly Disagree, Disagree, Neutral, Agree, Strongly Agree

What different types of practical skills did you learn?*

- *Free Text Answer*

Section 4 Appreciation of Anatomy:

You became more aware of anatomical variation*

- Strongly Disagree, Disagree, Neutral, Agree, Strongly Agree

You became more aware that much remains to be discovered in human anatomy*

- Strongly Disagree, Disagree, Neutral, Agree, Strongly Agree

There is still scope for impactful research in clinical anatomical research*

- Strongly Disagree, Disagree, Neutral, Agree, Strongly Agree

Section 5 Non-clinical Skills:

How did your project develop your interpersonal skills?*

- *Free Text Answer*

Your team-working skills were improved*

- Strongly Disagree, Disagree, Neutral, Agree, Strongly Agree

Your presentation skills were improved*

- Strongly Disagree, Disagree, Neutral, Agree, Strongly Agree

Your communication skills with non-experts improved*

- Strongly Disagree, Disagree, Neutral, Agree, Strongly Agree

Your communication skills with experts improved*

- Strongly Disagree, Disagree, Neutral, Agree, Strongly Agree

Your self-directed learning skills were improved*

- Strongly Disagree, Disagree, Neutral, Agree, Strongly Agree

During the project you were able to work with people with varying levels of experience/expertise*

- Yes, No

Did you travel to different parts of the country to gain clinical exposure?*

- Yes, No

If yes, what did you learn from this experience?

- *Free Text Answer*
