# Supplementary material for: Impact of Anatomical Research Projects for Medical Students: A Cross‐Sectional Survey of Academic and Professional Skills, Clinical Aspirations and Appreciation of Anatomy
Source: Clin Anat. 2025 Jan 19;38(3):347–54. doi: 10.1002/ca.24259 (PMC11925134; doi:10.1002/ca.24259)
Supplement: Supplementary file 3 — Data S3. [file CA-38-347-s004.docx]

Qualitative review of outcomes on completion of anatomy research projects at the University of Cambridge

## CHESREC Ethics Review Reference Number: **CHESREC.2023.ET.55.Sinha**

### Why is this research being conducted?

This is an independent project led by former Part II project students aiming to assess the impact of completing a Part II anatomical research project at the University of Cambridge on four domains: students’ academic skills, interpersonal skills, clinical aspirations, and their appreciation of anatomy. It shall take the form of an electronic questionnaire distributed to students who completed projects between academic years 2014-15 and 2022-23. This project is not part of any course requirement and is conducted due to an interest in the value of the anatomical research projects which have been undertaken.

The purpose of assessing this is to understand how previous students feel the project impacted these four domains and thus their perceived value of completing such a project. We anticipate that students would have found the project valuable in developing foundational skills needed for a career as an academic doctor. However this project will highlight specific ways in which the project has impacted them with reference to the above four domains. Furthermore, anonymised findings from this survey shall be shared with the course Programme Coordinator and may inform future changes in how anatomy projects are organised and run with the aim to optimising what students gain from them in response to feedback.

### Why have I been asked to take part?

### You have been invited because you have completed a Part II anatomy research project between academic years 2014-15 and 2022-23

### Do I have to take part?

### No. You can ask questions about the research before deciding whether or not to take part. If you do agree to take part, you may withdraw yourself from the study at any time, without giving a reason, and without negative consequences, by advising us of this decision. If you choose to withdraw from the study please contact the research lead (Dr Amil Sinha [sinhaamil@gmail.com](mailto:sinhaamil@gmail.com)) and CHESREC ([chesrec@admin.cam.ac.uk](mailto:chesrec@admin.cam.ac.uk)). Please note that anonymisation shall be prioritised and as a result, once a response is submitted it will not be possible to trace it back to the individual. Participants will be able to navigate within the survey to edit their responses while completing the survey but will be unable to make any alterations after submission.

### What will happen to me if I take part in the research?

If you are happy to take part in the research, you will be asked to complete an electronic survey. The survey should take approximately 5-10 minutes.

If you are still happy to take part, you should tick the box within the survey to provide consent. You will be unable to submit your response unless this is ticked.

### Are there any potential risks in taking part?

### The following risks are involved in taking part: breach of confidentiality.

### To reduce any potential risks, data will be stored in an encrypted file on a restricted-access Google Drive folder. This data will be pseudonymised, with each participant referred to by a unique number rather than by name. Personal information such as e-mail addresses or names shall not be accessible by anyone other than the principal investigator (Prof Cecilia Brassett).

### Are there any benefits in taking part?

There will be no direct or personal benefit to you from taking part in this research.

### What happens to the data I provide?

### The information you provide during the study is the **research data**. Any research data from which you can be identified, such as your name, is known as **personal data**.

### **Personal data** will be stored in an encrypted file on a restricted-access Google Drive folder. No personal data is collected as part of the survey. All data will be deleted 3 years after publication.

### **Research data** (including consent forms) will be stored for at least 3 years after publication or public release of the work of the research.

### The researchers will have access to the research data. Responsible members of the University of Cambridge may be given access to data for monitoring and/or audit of the research.

### Will the research be published?

We aim to publish the research in an academic journal. We also aim to present the anonymised data at national academic conferences.

### Who is funding the research?

No funding is required.

### Who has reviewed this study?

This study has been reviewed by the Cambridge Higher Education Research Ethics Committee (CHESREC) Reference Number: **CHESREC.2023.ET.55.Sinha**

### Who do I contact if I have a concern?

If you have a concern about any aspect of this study, please contact the Cambridge Higher Education Research Ethics Committee (CHESREC): ([chesrec@cctl.cam.ac.uk](mailto:chesrec@cctl.cam.ac.uk)) who will do their best to answer your query. If you remain unhappy or wish to make a formal complaint, please contact the Research Integrity office of the University of Cambridge: ([researchintegrity@admin.cam.ac.uk)](mailto:researchintegrity@admin.cam.ac.uk).

### Data protection

Information about your rights with respect to your personal data can be found here: <https://www.information-compliance.admin.cam.ac.uk/data-protection/research-participant-data>.

### Further information and contact details

If you would like to discuss the research with someone beforehand (or if you have questions afterwards), please contact:

Amil Sinha

FY2 Doctor, Sandwell and West Birmingham NHS Trust

Former Part II Anatomy student

[sinhaamil@gmail.com](mailto:sinhaamil@gmail.com)
