# Supplementary material for: Impact of Anatomical Research Projects for Medical Students: A Cross‐Sectional Survey of Academic and Professional Skills, Clinical Aspirations and Appreciation of Anatomy
Source: Clin Anat. 2025 Jan 19;38(3):347–54. doi: 10.1002/ca.24259 (PMC11925134; doi:10.1002/ca.24259)
Supplement: Supplementary file 4 — Data S4. [file CA-38-347-s002.docx]

Qualitative review of outcomes on completion of anatomy research projects at the University of Cambridge

## CHESREC Ethics Review Reference Number: **CHESREC.2023.ET.55.Sinha**

## Name of researcher: Amil Sinha

|  |  | *Please check* |
| --- | --- | --- |
| 1 | I confirm that I have read and understand the participation information sheet for the above study. I have had the opportunity to consider the information, ask questions and have had these answered satisfactorily. |  |
| 2 | I understand that my participation is voluntary and that I am free to withdraw at any point without any adverse consequences or penalty before submission to a conference/journal. If you choose to withdraw from the study please contact the lead researcher (Dr Amil Sinha [sinhaamil@gmail.com](mailto:sinhaamil@gmail.com)) and CHESREC ([chesrec@admin.cam.ac.uk](mailto:chesrec@admin.cam.ac.uk)). |  |
| 3 | I understand that this project has been reviewed by the Cambridge Higher Education Studies Research Ethics Committee (CHESREC) and I may contact them if I have any concerns about this research project. |  |
| 4 | I understand who will have access to personal data provided, how the data will be stored and what will happen to the data at the end of the project. |  |
| 5 | I understand how this research will be presented, written up and published. |  |
| 6 | I understand how to raise a concern or make a complaint. |  |
| 7 | I give permission to be quoted directly in research outputs anonymously |  |
| 8 | I agree to take part in the study |  |

If you consent to the above and have read the Participant Information Sheet, please confirm so by checking the box at the top of the survey. You will only be able to submit a response if this box is checked.



AMIL SINHA 27/6/23

_______

Name of person taking consent Date Signature
